# Fronto-parietal homotopy in resting-state functional connectivity predicts task-switching performance

**DOI:** 10.1007/s00429-021-02312-w

**Published:** 2021-06-09

**Authors:** Antonino Vallesi, Antonino Visalli, Zeus Gracia-Tabuenca, Vincenza Tarantino, Mariagrazia Capizzi, Sarael Alcauter, Dante Mantini, Lorenzo Pini

**Affiliations:** 1 Department of Neuroscience and Padova Neuroscience Center, University of Padova, Via Giustiniani 5, 35128 Padova, Italy; 2 IRCCS San Camillo Hospital, Venice, Italy; 3 Instituto de Neurobiología, Universidad Nacional Autónoma de México, Queretaro, Mexico; 4 Department of Psychology, Educational Science and Human Movement, University of Palermo, Palermo, Italy; 5 Université Paul Valéry Montpellier 3, EPSYLON EA 4556, 34000 Montpellier, France; 6 Research Centre for Motor Control and Neuroplasticity, KU Leuven, Leuven, Belgium

**Keywords:** Homotopy, Hemispheric asymmetries, Task-switching, Mixing costs, Executive functions, Resting-state fMRI

## Abstract

Homotopic functional connectivity reflects the degree of synchrony in spontaneous activity between homologous voxels in the two hemispheres. Previous studies have associated increased brain homotopy and decreased white matter integrity with performance decrements on different cognitive tasks across the life-span. Here, we correlated functional homotopy, both at the whole-brain level and specifically in fronto-parietal network nodes, with task-switching performance in young adults. Cue-to-target intervals (CTI: 300 vs. 1200 ms) were manipulated on a trial-by-trial basis to modulate cognitive demands and strategic control. We found that mixing costs, a measure of task-set maintenance and monitoring, were significantly correlated to homotopy in different nodes of the fronto-parietal network depending on CTI. In particular, mixing costs for short CTI trials were smaller with lower homotopy in the superior frontal gyrus, whereas mixing costs for long CTI trials were smaller with lower homotopy in the supramarginal gyrus. These results were specific to the fronto-parietal network, as similar voxel-wise analyses within a control language network did not yield significant correlations with behavior. These findings extend previous literature on the relationship between homotopy and cognitive performance to task-switching, and show a dissociable role of homotopy in different fronto-parietal nodes depending on task demands.

**Supplementary Information:**

The online version contains supplementary material available at 10.1007/s00429-021-02312-w.

## Introduction

Homotopic functional connectivity is defined as the degree of synchrony in the time course of spontaneous activity between the two cerebral hemispheres (Stark et al. 2008). The integration between the left and right hemispheres is a robust feature with a peculiar functional hierarchy, with homotopy being highest in sensory-motor regions and lowest in higher-level associative regions including the prefrontal cortex (PFC) (Stark et al. 2008; Zuo et al. 2010). This general gradient is related to structural connections of the brain. The corpus callosum, the major inter-hemispheric connectivity tract, plays a key role in mediating homotopy functional integration (for a recent review see Jin et al. 2020). This assumption is supported by studies reporting an association between homotopic connectivity dysfunction and degraded corpus callosum integrity, which is accompanied by lower processing speed and worse cognitive performance, as shown in cognitive aging (Persson et al. 2006; Sullivan et al. 2006; Madden et al. 2009; Gorbach et al. 2017; Avelar-Pereira et al. 2020).

Moreover, functional homotopy is influenced by many alterations of brain physiology (Jin et al. 2020). Global functional homotopy follows a quadratic trend across the lifespan, with a progressive decrease during development, conceivably accompanying functional specialization, and a later increase with cognitive aging (Kelly et al. 2009; Supekar et al. 2009; Zuo et al. 2010). An age-related increase in homotopy in the PFC has been shown to be associated with a worsening of working memory performance over 5 years in the adult lifespan (Avelar-Pereira et al. 2020). Homotopic alterations have been also reported in several brain pathological conditions, such as psychiatry diseases, stroke, and epilepsy (Mancuso et al. 2019).

To date, few studies assessed a significant correlation between human cognitive abilities and homotopy, such as visuo-spatial attention in adolescence (Gracia-Tabuenca et al. 2018). The association between homotopic connectivity and cognitive control has been less investigated in younger adults. Unraveling the role between homotopic brain integration and high-level cognitive functions in healthy adults will help to clarify the functional meaning of homotopy.

In the present study, we specifically focused on task-switching (Rogers and Monsell 1995; Rubin and Meiran 2005), a well-validated paradigm that provides two complementary indices of cognitive control: mixing costs, that is, response time (RT) difference between repeat trials in task-switching and single task-blocks; and switching costs, that is, RT difference between switch and repeat trials during the task-switching blocks.

Previous studies associated higher integrity in the genu of the corpus callosum with better performance in task-switching in both younger and older adults (Gold et al. 2010; Vallesi et al. 2016). Specifically, higher fractional anisotropy and lower mean diffusivity in this white matter tract in younger adults predicted lower mixing costs, whereas no correlations were found for the switching costs (Vallesi et al. 2016). Mixing costs have been interpreted as a measure of sustained control processes, including maintenance and monitoring of task-set, or management of competition between task-sets in task-switching blocks (Ilan and Miller 1994; Braver et al. 2003; Rubin and Meiran 2005; cf. Los 1996). No correlations were instead found for the switching costs, a measure of phasic processes such as task-set reconfiguration, updating, or interference (Rogers and Monsell 1995; Allport and Wylie 1999). These studies suggest a dissociation between these two types of costs (also see Ambrosini et al. 2019, for factor analysis evidence).

Concerning the neural basis of task-switching, neuroimaging studies have shown that regions that belong to the fronto-parietal network (FPN) are activated during the performance of different types of task-switching (e.g., Sohn et al. 2000; Kim et al. 2011; Jamadar et al. 2015; Vallesi et al. 2015). More specifically, animal and human neuroscience research provided evidence that lateral prefrontal regions are involved in representing task-sets (Miller and Cohen 2001; Sakai 2008), whereas dorsal parietal regions are important to voluntarily shift attentional focus as well as planning and implementing task-relevant selection of stimuli and responses (Posner and Petersen 1990; Corbetta and Shulman 2002; Corbetta et al. 2008; Cabeza et al. 2008).

Compatible with this view, a recent Transcranial Magnetic Stimulation (TMS) study on task-switching (Muhle-Karbe et al. 2014) showed that inhibition of the left inferior frontal junction may interfere with task goal updating, whereas inhibition of the left intra-parietal sulcus may disrupt the ability to update the specific response sets. This suggests that the intra-parietal sulcus is more involved in translating abstract task goals into specific action rules to guide task implementation.

Regarding the two behavioral costs, previous studies have associated mixing and switching costs with the functioning of right and left-lateralized prefrontal regions, respectively. For example, Braver and colleagues (2003) found that faster repeat-trial responses in mixed blocks were associated with higher right anterior prefrontal activations, whereas faster switching responses were associated with higher left parietal activations. Another resting-state EEG study using three different task-switching paradigms (Ambrosini and Vallesi 2016) showed that right-ward asymmetrical activity (operationalized as β/α ratio) in a dorsolateral prefrontal source predicted smaller mixing costs, while left-ward asymmetrical activity in the same region predicted smaller switching costs, compatibly with a role of these lateralized activities in complementary executive functions (Vallesi 2021).

In the light of the previous literature, we investigated in this study whether global brain homotopy and more specific homotopy in nodes of the FPN could predict cognitive performance in a classical task-switching paradigm. We analyzed mixing and switching costs as markers of sustained and phasic control processes, respectively. We also manipulated cue-to-target interval (CTI: 300 vs. 1200 ms) on a trial-by-trial basis to differentially modulate cognitive demands in this paradigm (e.g., Meiran 1996; Cooper et al. 2015; Capizzi et al. 2020).

As commonly observed in the task-switching literature (see Kiesel et al. 2010; Karayanidis and Jamadar 2015, for reviews), given that our CTI manipulation was trial-by-trial, we expected a sub-optimal task-preparation (and thus worse performance) for the short CTI, as in this situation the maximum level of preparation has been shown to be timed later for shorter CTIs than for longer ones (Altmann and Gray 2008). Moreover, sub-optimal preparation for short CTIs would be more generally exacerbated by variable foreperiod effects, occurring when short and long preparatory intervals are randomly intermixed within a block of trials (e.g., Niemi and Näätänen 1981; Vallesi 2010).

Additionally, in terms of brain-behavior correlations, we also expected task-switching behavioral performance to be worse with higher brain functional homotopy, specifically in regions belonging to the FPN, with possibly differential effects of switching and mixing costs with short and long CTIs. Nevertheless, the opposite prediction could also be put forward, as previous studies have shown that more complex and difficult tasks (e.g., task-switching) are associated with higher interhemispheric interactions especially in fronto-parietal regions (Banich and Belger 1990; Ocklenburg et al. 2012). This alternative prediction is, however, much less likely, as task demands are conceivably low during the resting-state period in which functional homotopy is computed, and it may not be related to task-related inter-hemispheric functional integration required for demanding tasks. We further investigated cognitive-functional coupling using network connectivity strength, a functional measure investigating brain integration between regions without considering brain interhemispheric interactions.

## Methods

### Participants

Forty-seven young healthy individuals (26 females; mean age = 24.7 years, SD = 3.5) voluntarily took part in the study. All participants gave informed consent prior to their recruitment. For their time, they were reimbursed for both the fMRI and the behavioral experimental sessions (see details on the *General procedure* section). All of them were right-handed as assessed with the Edinburgh Handedness Inventory (Oldfield 1971) with an average score of 87.8 (SD = 12.2), reported no history of neurological or psychiatric disorders, had a normal color vision and normal or corrected-to-normal visual acuity. The procedures involved in this study were approved by the Bioethical Committee of the Azienda Ospedaliera di Padova–AOP (Prot # 2758P).

### General procedure

Each participant was tested in two separated experimental sessions (mean inter-session interval = 21.5 days, SD = 17.4). In the first session, structural and resting-state functional MRI (rs-fMRI) data were acquired along with some task-related fMRI data collected for different aims and published elsewhere (e.g., Visalli et al. 2019). The order of acquisition was: (i) rs-fMRI, (ii) task-related fMRI, and (iii) structural MRI. In the second session, participants performed the color-shape task-switching paradigm, which is the focus of the present study.

### The color-shape task-switching paradigm

**Fig. 1:**
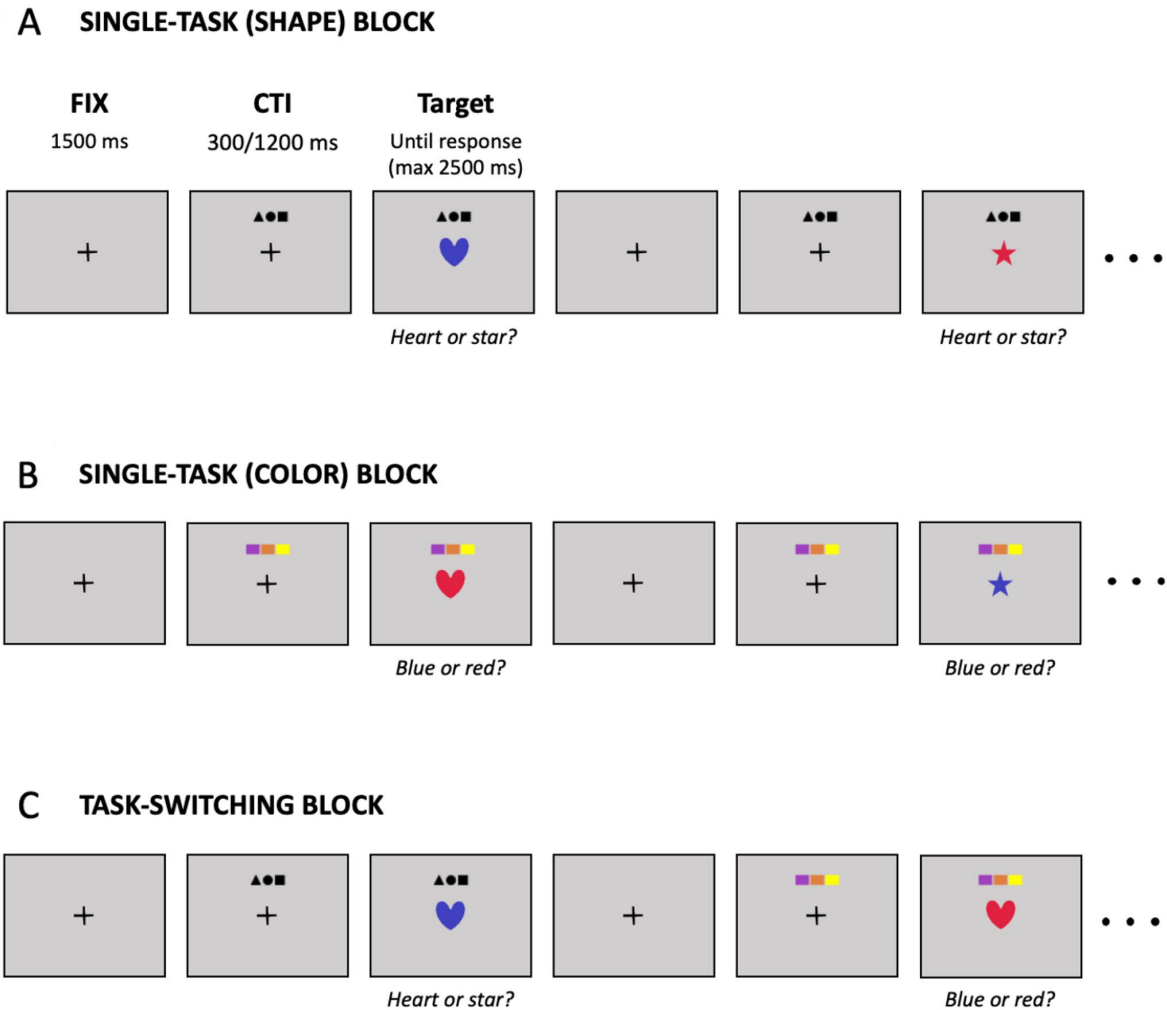
Task-switching paradigm. Trials started with a black fixation cross. After 1500 ms, a cue stimulus signaled the task to be performed: three colored rectangles required the participant to indicate the color of the target (blue or red); three small black shapes required the participant to indicate the shape of the target (heart or star). After a cue-to-target interval (CTI) of either 300 or 1200 ms the target was displayed until the participant’s response (max 2500 ms). Participants completed eight blocks of trials of two different types: single-task and task switching blocks. In single-task blocks participants were required to perform only one task during the entire block, either shape (**A**) or color (**B**). In task-switching blocks (**C**) participants were required to indicate either the shape or the color of the target according to the cue. The structure of the trial was the same in all block types

Participants performed a color-shape task-switching paradigm (Rubin and Meiran 2005; Ambrosini and Vallesi 2016; Vallesi et al. 2016), which was implemented in MATLAB (The MathWorks, Inc., Natick, Massachusetts, United States) using the Psychophysics Toolbox 3 (Kleiner et al. 2007). A graphical representation of the paradigm is presented in Fig. 1. Participants sat in a dimly lit soundproof cabin at a viewing distance of approximately 60 cm from the computer screen. Each trial started with a black fixation cross (visual angle: 0.28° by 0.28°) displayed at the center of the screen. After 1500 ms, a cue stimulus (visual angle: 5.5° by 1.7°) signaling the task to be performed was presented 4.6° above the fixation cross. The color task cue consisted of a row of three colored rectangles (purple, orange, and yellow), while the shape task cue consisted of a row of three small black shapes (a triangle, a circle, and a square). Graphic cues were chosen to limit the use of linguistic information (Ambrosini and Vallesi 2016). After a cue-to-target interval (CTI) of either 300 ms (short CTI) or 1200 ms (long CTI), the fixation cross was replaced by the target stimulus consisting of a heart or a star shape (visual angle: 3.5° by 3.5°) in either red or blue color. The short CTI was long enough for task-cue encoding but it was probably too short for task reconfiguration (cf., Meiran 1996; see also: Luria and Meiran 2006; Schneider 2016; Lange et al. 2018). The long CTI was instead long enough for the completion of task reconfiguration since its duration was considerably higher than average RTs in slow conditions (Rogers and Monsell 1995; Meiran 1996). The target was displayed until a response was produced by the participant (maximum response time allowed: 2500 ms). Participants were instructed to respond as fast and accurately as possible to either the shape or the color of the target on the basis of the target cue. Responses were provided by pressing one of the two lower buttons of the CEDRUS RB-840 response pad with the left and right index fingers. The assignment of the two shapes and the two colors to response keys (2-by-2 possible combinations) was counterbalanced across participants. The four possible stimuli (two colors by two shapes combination) and the two CTIs were pseudo-randomly interleaved within each block of trials ensuring that each possible stimulus-by-CTI combination was presented an equal number of times across trial types (see below).

Participants completed eight blocks of trials of two different types: (i) single-task blocks, during which participants performed only one task for the entire block (either color or shape), (ii) and task-switching blocks, in which participants were required to switch or repeat the task performed at the previous trial on the basis of the task cue. In the single-task blocks, the cue was always the same (i.e., the color task cue for the pure color block, and the shape task cue for the pure shape block). The task procedure was structured as follows: (i) two single-task blocks of 40 trials each (one for each task; task order counterbalanced across participants); (ii) two task-switching blocks of 65 trials each (32 switch trials + 32 repeat trials + the initial trial); two 40-trial single-task blocks (task order inverted with respect to the two initial single-task blocks). The CTI manipulation was implemented in each block type. This trial-wise (instead of block-wise) CTI manipulation was used to overcome the possible confounding effects due to changes in task strategy or differences in memory load (Meiran 1996). The first three blocks were preceded by a short practice phase (8 trials for the single-task blocks, 16 trials for the switching block). During practice only, feedback about accuracy and speed was provided after each response (the Italian translations for “Well done”, “Correct, but try to be faster”, or “Wrong” were displayed at the center of the screen after a correct response given within the 2 s maximum RT allowed in test trials, a correct response > 2 s, or an incorrect response, respectively). Self-paced breaks were available between blocks.

### Behavioral scores

**Fig. 2:**
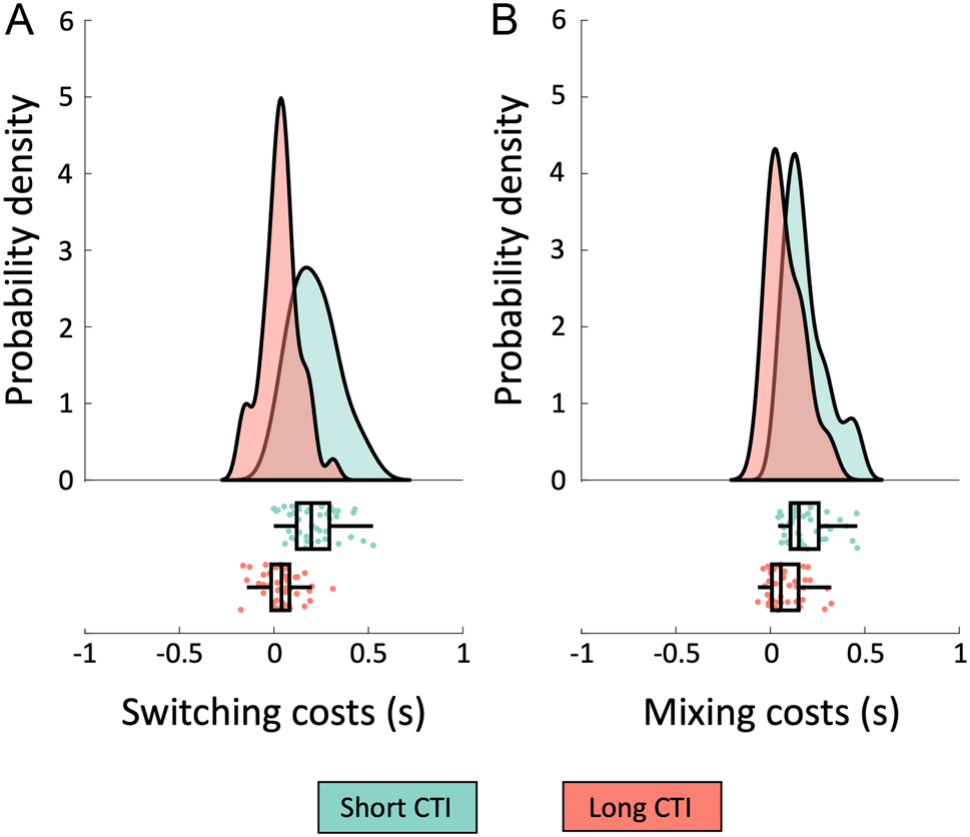
Raincloud plots of behavioral costs. The panels show the distribution in our sample of switching (**A**) and mixing (**B**) costs at short (300 ms) and long (1200 ms) CTIs. Data points in the lower panels represent individual costs, which are overlaid with boxplots displaying sample median alongside interquartile range. The raincloud plots were generated using codes provided by Allen et al. (2019)

Data from three participants were discarded since they were excluded for imaging issues (see below). The final sample for all the reported analyses, hence, included 44 participants. A sensitivity power analysis (G*Power 3 software; Faul et al. 2007) revealed that our sample size was large enough to detect significant (*α* = 0.05) mean differences between two dependent means (e.g., the main effect or the interaction effect of a 2-by-2 repeated measures ANOVA) with a medium effect size *d* = 0.43 (Cohen 1988) with a statistical power of 0.80. Data from the practice phase, first trial in a block, error (incorrect or no response) and post-error trials were excluded from analysis. For each trial type (i.e., single-task, repeat and switch trials) at each CTI, response time (RT) values more extreme than one and a half times the interquartile range (i.e., the difference between the upper and lower quartile) above the upper quartile or below the lower quartile were identified as outliers (Borcard et al. 2011) and excluded (mean excluded trials = 5.1%, SD = 1.9%). Mean RTs were, then, computed for each trial type at each CTI and used to calculate two behavioral cost measures: (i) switching costs (Monsell 2003), computed as the difference between the mean RT from switch trials and that from repeat trials; (ii) and mixing costs (Rubin and Meiran 2005), computed as the difference between the mean RT from repeat trials and that from pure trials. The resulting four scores (i.e., mixing and switching costs at short and long CTI; Fig. 2) were used for the brain-behavior correlations with the homotopy scores. The correlation between switching and mixing costs was *r* = 0.03 (*p* = 0.858) at short CTI, and *r* = − 0.40 (*p* = 0.005) at long CTI.

### MRI acquisition

Structural and functional images were acquired with a 3 T Ingenia Philips scanner (Philips Medical Systems, Best, The Netherlands) at the Neuroradiology Unit of the Padova University Hospital. The system was equipped with a 32-channel head-coil. Functional data consisted of 250 T2*-weighted echo-planar image (EPI) volumes (repetition time, TR: 2000 ms; echo time, TE: 30 ms; 39 axial-slices with ascending acquisition; voxel size: 3 × 3 × 3 mm; flip angle, FA: 76°; acquisition matrix: 84 × 84). After the functional session, high resolution T1-weighted anatomical images were acquired (TR/TE: 8.10/3.72 ms; 180 sagittal slices; voxel size: 1 × 1 × 1 mm; FA: 8; acquisition matrix: 256 × 256). To reduce head movements during data acquisition, small foam cushions and sponge pads were placed around the participant’s head.

### Resting-state fMRI preprocessing

Two participants were excluded after visual quality check, reporting enlarged ventricles which could affect normalization steps, while one participant was excluded due to an incomplete rs-fMRI exam, thus leaving a sample of 44 participants for the analysis. Functional data were preprocessed using the FMRIB Software Library, version 6.0.0) (Smith et al. 2004), according to a previously described pipeline (Pini et al. 2020). Functional data were motion corrected, brain extracted and registered to the corresponding structural image with rigid-body transformation and nonlinearly registered to a symmetric brain template (ICBM 2009a Nonlinear symmetric template). Functional data were high-pass filtered (100 s) and for each functional volume, the framewise displacement (FD) was estimated and volumes with more than 0.25 mm FD were regressed out from the time series. Confounding variables also included the average signal of white matter and cerebro-spinal fluid, 6 head motion parameters, the derivatives of these 8 regressors, and the square of these 16 regressors, as suggested by Satterthwaite et al. (2013). In addition, the time-courses of the first 5 principal components calculated over white matter and cerebro-spinal fluid voxels were included as confounding variables to minimize further the effect of physiological noise, a method referred as *aCompCor,* widely used in rs-fMRI pre-processing (Behzadi et al. 2007; Chai et al. 2012). Finally, images were spatially smoothed using a Gaussian kernel with a full-width at half-maximum of 6 mm.

### Fronto-parietal network identification

**Fig. 3:**
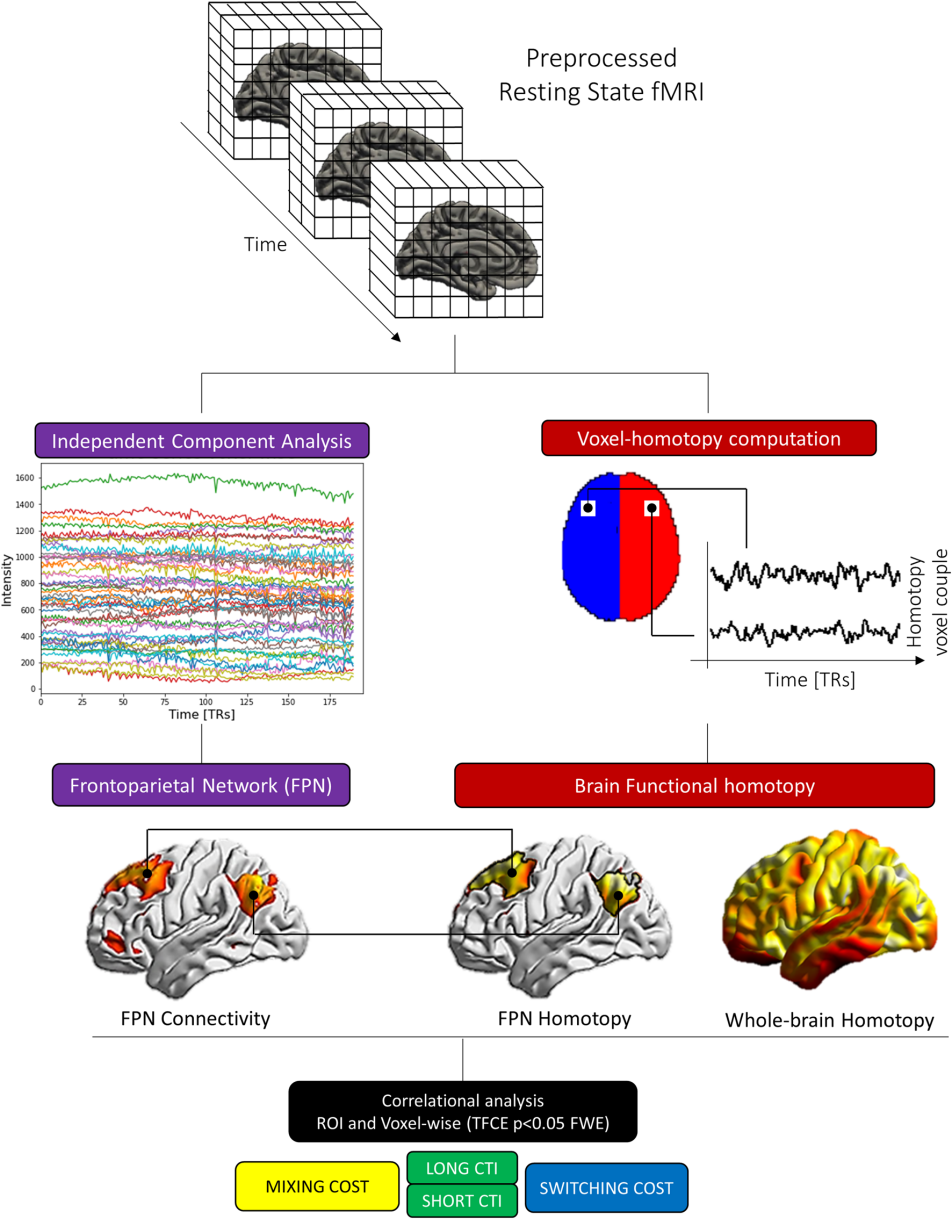
Workflow of the functional connectivity and homotopy analysis. From the rs-fMRI preprocessed data we extracted the frontoparietal network (FPN) through an independent component analysis approach, and voxel-mirror homotopy properties. Functional maps were then correlated with behavioral performance (mixing and switching costs) through a permutation strategy

For an overview of the methodology see Fig. 3. First, we identify brain regions organized within the FPN through an independent component analysis (ICA). In this step, we further included 15 participants with the same rs-fMRI data who did not come back to complete the second (behavioral) session and an independent dataset of 21 healthy young individuals with MRI sequences with equivalent acquisition parameters collected at a different site (see Supplementary Methods), for a total of 83 healthy participants (45 females; mean age = 24.4 years, SD = 3.2; EHI = 84.5, SD = 27.2). The inclusion of a larger sample is known to ensure a more robust output in fMRI analyses (Desmond and Glover 2002; Mumford and Nichols 2008; Lindquist et al. 2013). Preprocessed fMRI data were fed into the Group ICA Toolbox (GIFT version 3.0a http://mialab.mrn.org/software/gift/) (Calhoun et al. 2001). The number of independent components extracted (*n* = 76) was chosen according to the minimum description length criteria (Li et al. 2007). The resulting FPN group map was identified through a template matching spatial correlation procedure with standard templates (Shirer et al. 2012), and visually inspected to exclude components underlying artifacts, according to Griffanti et al. (2017) criteria. The FPN map was binarized at *z* > 2 and used as a whole region of interest (ROI) in the homotopy analysis, according to previous procedures applied by our group to investigate this network (Pini et al. 2020). For simplicity, results are reported in the left hemisphere. Finally, we included the language network, identified through a spatial correlation procedure with the *fslcc* utility from FSL compared with the Shirer’s language template (Shirer et al. 2012). This network was included as a control network. We assumed that homotopy of regions overlapping with language network hubs would not exhibit a significant association with executive tasks, since these regions converge on a left-lateralized network active during speech reception and production (Braga et al. 2020).

Finally, for each participant we computed the correlation between cognitive performance and FPN functional connectivity strength. Specifically, for each participant, the GIFT FPN maps were inserted into a voxel-wise correlation analysis with the switching and mixing tasks. Finally, to confirm voxel-wise correlation, FPN-ROI analysis was performed, computing the correlation between *z* score mean FPN strength (i.e., mean FPN values—which express the degree of functional connectivity within the network for each participant) and cognitive performance.

### Voxel-mirror homotopy computation

For each participant, we computed homotopy brain property as the voxel-mirrored homotopic functional connectivity between every pair of mirror voxels through in-house scripts based on R using unsmoothed data as previously reported (Gracia-Tabuenca et al. 2018; Zuo et al. 2010). Briefly, in the symmetrical brain space we computed the Pearson’s correlation coefficient between each voxel time-series residuals (after pre-processing steps) with the hemispheric counterpart. Voxels belonging to the three sagittal midline slices (9 mm) were excluded, to avoid inter-hemispheric partial volume effect. Then correlation values were Fisher *Z*-transformed. Finally, homotopy maps of each individual were smoothed with a gaussian kernel of 6 mm.

We included in the analysis both whole-brain homotopy maps (whole voxel values) and homotopy connectivity within the FPN, computed masking the whole-brain homotopy maps with the group ROI-FPN binarized map (see previous section).

### Brain functional-behavioral score correlations

We investigated the association between brain functional properties (i.e., connectivity strength and homotopy) and mixing/switching costs in different CTI (long, short) in the sample of participants who completed both fMRI and behavioral sessions.

This analysis was performed at FPN ROI levels: (i) for the homotopy analysis, we first averaged homotopy values of voxels within the FPN map. Then we computed correlational analysis between behavioral measures and mean homotopy; (ii) for the functional connectivity strength analysis, the FPN maps from GIFT were threshold at *z* > 2 and an average score for each participant was calculated. The ROI analysis was repeated considering whole (left) brain mean homotopy values to assess whether the association between homotopy and mixing/switching costs was stronger within FPN regions than for the whole brain. Due to the non-linear distribution of homotopy values, we performed a nonparametric Spearman correlation to assess the association between connectivity organization and cognition. *p* values were corrected for multiple comparisons (*n* = 4 behavioral measures; *p* < 0.0125) to control for type I error with Bonferroni correction.

At the voxel-wise level, we investigated brain-behavior correlation between cognitive performance with both FPN connectivity and homotopy strength. We implemented a nonparametric inference based on permutations (*n* = 5000) using FSL *randomise* (https://fsl.fmrib.ox.ac.uk/fsl/fslwiki/Randomise). Multiple comparisons were corrected across space using familywise error (FWE) based on permutation testing at a threshold-free cluster enhancement (TFCE; Smith and Nichols 2009). Positive and negative associations between homotopy and behavioral measures were tested at a TFCE level of *p* < 0.025 corresponding to a two-tailed *p* < 0.05 (*randomise* correction is only performed on one-tailed tests). Each contrast was restricted to the FPN map, computed as described above.

### Reliability analysis

To ensure that the mean homotopy map used in the correlational analysis exhibits a reliable profile, we computed, through the same procedure, voxel-mirrored homotopic functional connectivity from a larger open-source available dataset. To this aim, we retrieved participants from the Population Imaging of Psychology dataset (PIOP2), which is released within the Amsterdam Open MRI Collection (AOMIC). This dataset was selected according to the number of scans (250 scans) and TR (2000 ms), which were comparable with the dataset used in the present study (240 scans and TR = 2000 ms). For further details about PIOP2 dataset, see Snoek et al. (2021). From the PIOP2 dataset, we retrieved 224 participants with both structural MRI and rs-fMRI data available. The list of PIOP2 participants included in this analysis is reported in Supplementary Table S1. From the PIOP2 dataset, we computed both mean *Z*-Fisher homotopy maps and one-sample homotopy *t*-maps. We then ran spatial cross-correlation between both mean and t-maps from PIOP2 and the study dataset through the FSL utility fslcc (FSL v.6.0.0; https://fsl.fmrib.ox.ac.uk/fsl/).

Reliability of behavioral measures—i.e., mean RT for each trial type (pure, switch, and repeat trials) at each CTI (300 and 1200 ms) and the resulting mixing and switching costs—was evaluated by means of split-half correlations corrected with Spearman-Brown formula. Reliability coefficients were calculated as follows: (i) trials of each participant for each trial type-by-CTI combination were randomly splitted into two subsets of equal size; (ii) mean RTs was computed separately for the two subsets; (iii) switching and mixing costs were also calculated for the two subsets; (iv) correlations of participant’s mean RTs and costs between the two subsets were calculated and corrected. The procedure was repeated 2000 times and the median correlation taken as an index of reliability.

## Results

### Behavioral results

**Fig. 4:**
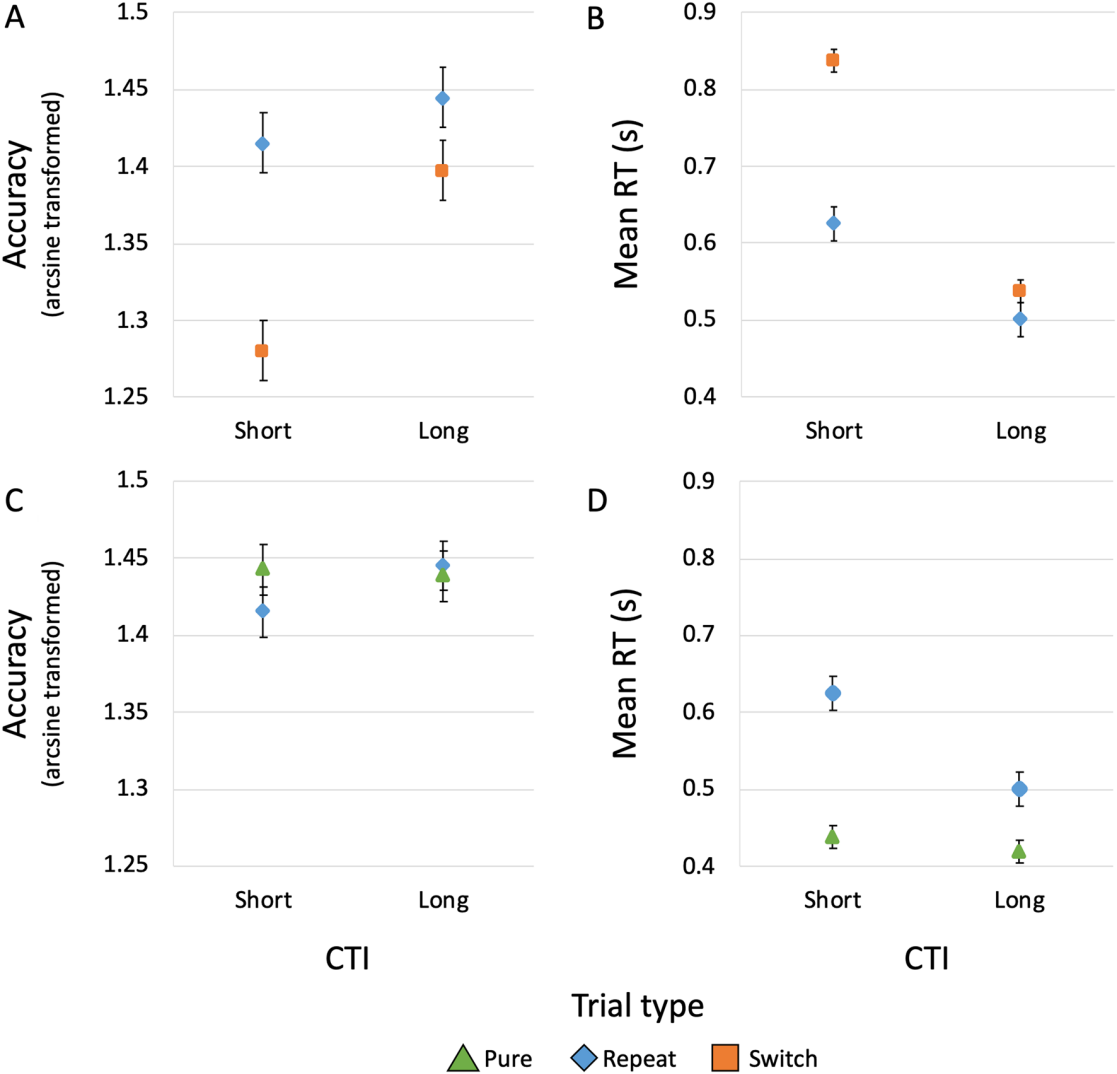
Trial type by CTI interaction plots. **A** Marginal means of arcsine transformed accuracy rates for repeat (blue diamonds) and switch trials (orange squares) at the short (i.e., 300 ms) and long (i.e., 1200 ms) cue-to-target intervals (CTI). **B** Marginal means of mean response times (RT) for repeat and switch trials at the short and long CTI. **C** Marginal means of arcsine transformed accuracy rates for pure (green triangles) and repeat trials (blue diamonds) at the short and long CTI. **D** Marginal means of mean RT for pure and repeat trials at the short and long CTI. Error bars represent the standard error of the mean

Descriptive statistics about switching and mixing costs are shown in Fig. 4 and Table 1. Concerning switching costs, 2-by-2 repeated-measures ANOVAs with within-subject factors “Trial type” (levels: repeat and switch trials) and CTI (levels: short and long) were conducted on (arcsine transformed) accuracy rates and mean RTs. The ANOVA on accuracy (see Fig. 4, Panel A) showed significant effects for Trial type (*F*(1,43) = 26.2, *p* < 0.001, partial *η*^2^ = 0.38), CTI (*F*(1,43) = 25.5, *p* < 0.001, partial *η*^2^ = 0.37), and their interaction (*F*(1,43) = 9.46, *p* = 0.004, partial *η*^2^ = 0.18). Accuracy was higher for repeat trials at long compared to short CTI, although this CTI effect was greater for switch trials. Post-hoc comparisons (Holm correction method) showed a significant trial-type difference at short CTI (mean difference = 0.14, SE = 0.02, *t* = 5.92, *p* < 0.001, 95% CI for mean difference = 0.07–0.20, Cohen’s *d* = 0.89), but not at long CTI (mean difference = 0.05, SE = 0.02, *t* = 2.10, *p* = 0.118, 95% CI = − 0.01–0.11, Cohen’s *d* = 0.32). They also showed a significant CTI difference for switch trials (mean difference = − 0.12, SE = 0.02, *t* = − 5.76, *p* < 0.001, 95% CI = − 0.17 to − 0.06, Cohen’s *d* = -− 0.87), but not for repeat trials (mean difference = − 0.03, SE = 0.02, *t* = − 1.46, *p* = 0.296, 95% CI = − 0.08–0.02, Cohen’s *d* = − 0.22). The ANOVA on RTs revealed significant effects for Trial type (*F*(1,43) = 108.42, *p* < 0.001, partial *η*^2^ = 0.72), CTI (*F*(1,43) = 291.61, *p* < 0.001, partial *η*^2^ = 0.87), and their interaction (*F*(1,43) = 52.30, *p* < 0.001, partial *η*^2^ = 0.55). Figure 4, Panel B shows that RTs were longer in switch trials. They were longer also at the short CTI and this CTI effect was greater for switch trials. Post-hoc comparisons revealed that CTI differences were significant in both repeat trials (mean difference = 0.12 s, SE = 0.02, *t* = 7.13, *p* < 0.001, 95% CI = 0.08–0.17, Cohen’s *d* = 1.08) and switch trials (mean difference = 0.30 s, SE = 0.02, *t* = 17.25, *p* < 0.001, 95% CI = 0.25–0.35, Cohen’s *d* = 2.60). Trial type differences were significant at both short (mean difference = − 0.21 s, SE = 0.02, *t* = − 12.45, *p* < 0.001, 95% CI = − 0.25 to – 0.17, Cohen’s *d* = − 1.88) and long CTI (mean difference = − 0.03 s, SE = 0.02, *t* = − 2.11, *p* = 0.037, 95% CI = − 0.08–0.01, Cohen’s *d* = − 0.32). A paired samples *t* test comparing switching costs at short vs long CTI revealed a significant difference (mean difference = 0.18, SE = 0.03, *t*(43) = 7.23, *p* < 0.001, 95% CI = 0.13–0.23, Cohen’s *d* = 1.09).

**Table 1:** Descriptive statistics of behavioral data

Trial type	CTI (ms)	RT (ms)		Accuracy (%)	
		Mean	SD	Mean	SD
Pure	300	439	48	97.9	1.7
	1200	420	54	97.5	2.3
Repeat	300	625	135	95.8	4.7
	1200	501	123	97.1	3.2
Switch	300	838	189	90.5	6.3
	1200	537	123	95.4	4.5

*CTI*  cue-to-target interval

Concerning mixing costs, 2-by-2 repeated-measures ANOVAs with within-subject factors “Trial type” (levels: pure and repeat trials) and CTI (levels: short and long) were conducted on (arcsine transformed) accuracy rates and mean RTs. The ANOVA on accuracy (see Fig. 4, Panel C) showed no significant effects for Trial type (*F*(1,43) = 0.35, *p* = 0.560, partial *η*^2^ = 0.008), CTI (*F*(1,43) = 1.08, *p* = 0.305, partial *η*^2^ = 0.02), and their interaction (*F*(1,43) = 2.34, *p* = 0.134, partial *η*^2^ = 0.05). The ANOVA on RTs revealed significant effects for Trial type (*F*(1,43) = 90.08, *p* < 0.001, partial *η*^2^ = 0.68), CTI (*F*(1,43) = 92.70, *p* < 0.001, partial *η*^2^ = 0.68), and their interaction (*F*(1,43) = 56.82, *p* < 0.001, partial *η*^2^ = 0.57). As shown in Fig. 4, Panel D, RTs were shorter in pure trials. Moreover, they were also shorter with long CTI, especially in repeat trials. Post-hoc comparisons showed that the CTI differences were significant for repeat trials (mean difference = 0.12 s, SE = 0.01, *t* = 12.18, *p* < 0.001, 95% CI = 0.10–0.15, Cohen’s *d* = 1.84), but not for pure trials (mean difference = 0.02 s, SE = 0.01, *t* = 1.88, *p* < 0.063, 95% CI = − 0.01–0.05, Cohen’s *d* = 0.28). Trial type differences were significant at both short (mean difference = − 0.19 s, SE = 0.02, *t* = − 11.85, *p* < 0.001, 95% CI = − 0.23 to – 0.14, Cohen’s *d* = − 1.79) and long CTIs (mean difference = − 0.08, SE = 0.02, *t* = − 5.16, *p* < 0.001, 95% CI = − 0.12 to − 0.04, Cohen’s *d* = − 0.78). A paired samples *t* test comparing mixing costs at short vs long CTI revealed a significant difference (mean difference = 0.11, SE = 0.01, *t*(43) = 7.54, *p* < 0.001, 95% CI = 0.08–0.13, Cohen’s *d* = 1.14).

### Homotopy map results

**Fig. 5:**
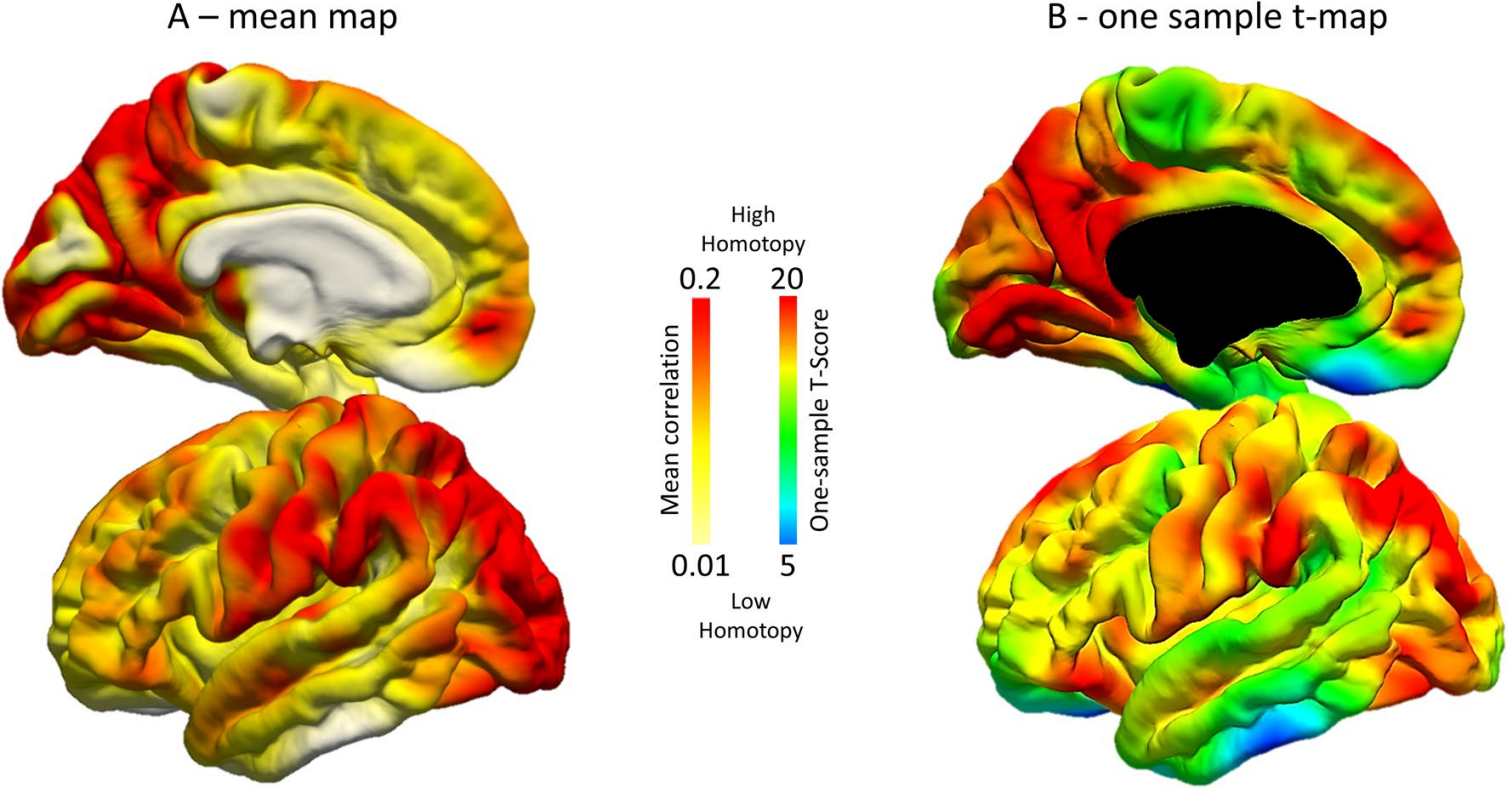
Homotopy maps in the whole sample of participants (*n* = 83, only 44 of whom then performed the task-switching test). Mean *Z* Fisher maps (**A**) and one-sample *t* test (**B**) are reported on the left fsaverage surface. Red colors: higher homotopy functional connectivity

Mean maps of the larger sample (*n* = 83) showed higher homotopy values within the motor and visual regions, and a decreased pattern in medial frontal, orbitofrontal and limbic regions (Fig. 5, Panel A). This pattern was echoed by the one-sample *t* map (Fig. 5, Panel B) in the whole sample (fsl-randomize, *n* = 5000 permutation). *T* values highlight consistent homotopy values across individuals, incorporating inter-subject variability. Moreover, the same pattern was reported in the subsample of participants who performed the cognitive tasks (*n* = 44) (Supplementary Figure S1), supporting the robustness of homotopy findings in our sample.

### Homotopy association with cognitive tasks

**Fig. 6:**
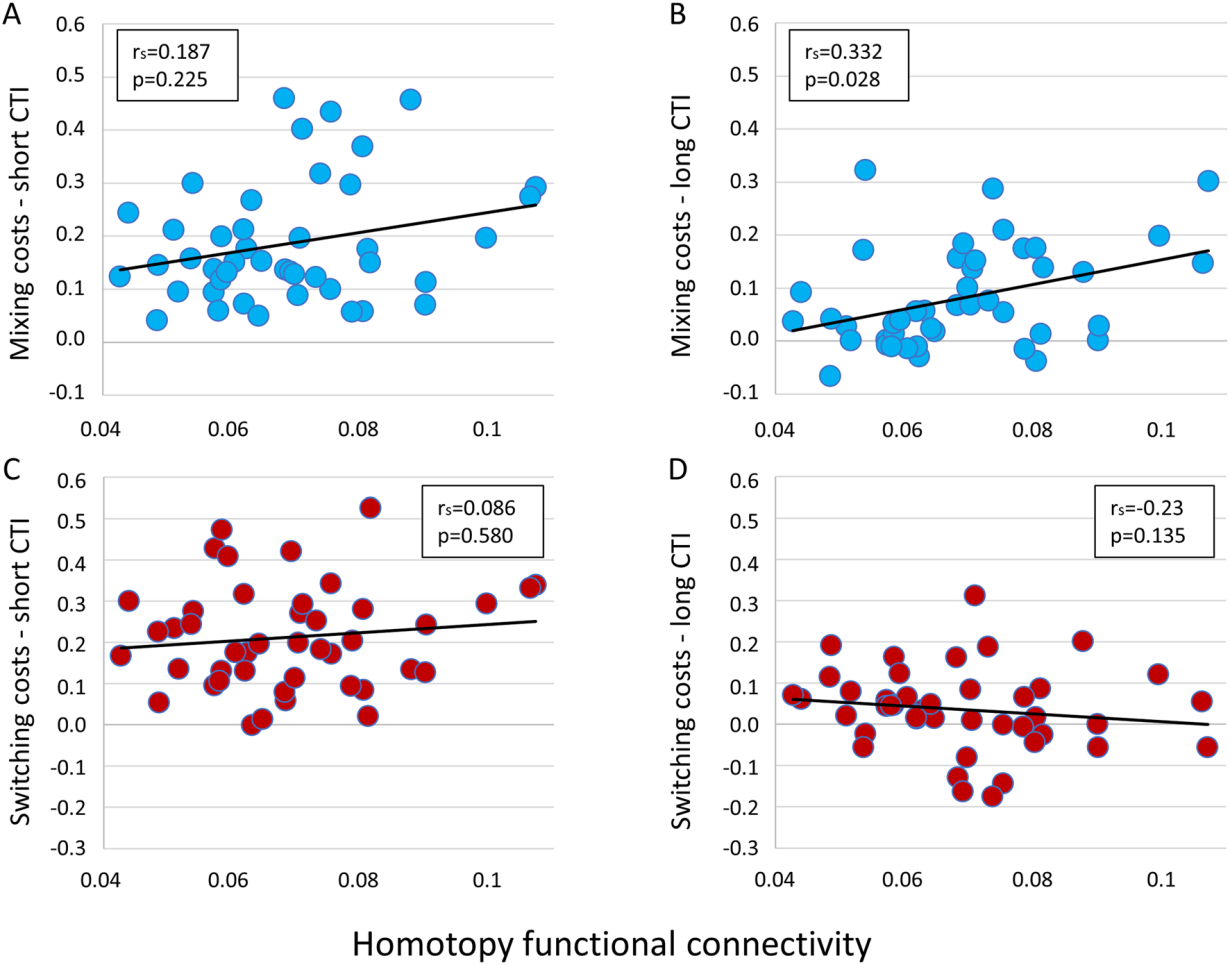
Correlation between behavioral measures and whole brain average homotopy. *r*_s_ = nonparametric Spearman correlations. Panels **A** and **B** show this correlation for mixing costs, short and long Cue-to-Target intervals (CTI), respectively; Panels **C** and **D** show the correlation for the switching costs, short and long CTI, respectively

There was a positive correlation between homotopy values at the whole-brain level and mixing costs with long CTI duration (Fig. 6, Panel B), although not surviving multiple comparison correction (Spearman *r*, *r*_s_ = 0.332; *p* < 0.028). The association between whole-brain homotopy and mixing costs with short CTI and switching costs (both long and short CTI) showed no significant associations (*p* > 0.1; Fig. 6).

**Fig. 7:**
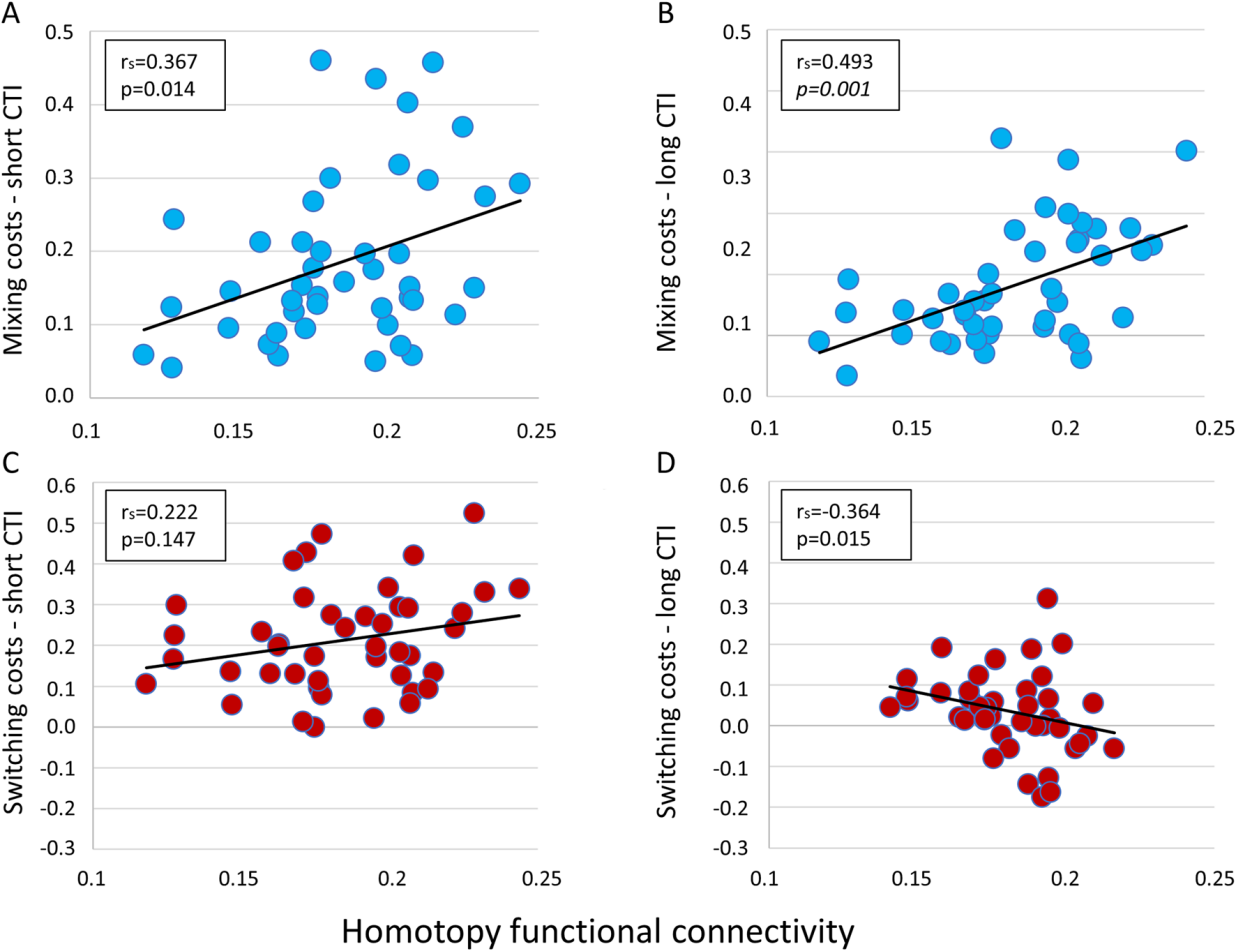
Correlation between behavioral measures and fronto-parietal homotopy; *r*_s_ = nonparametric Spearman correlations. *p* values surviving multiple comparisons are reported in italics. Panels **A** and **B** show this correlation for mixing costs, short and long CTI, respectively; Panels **C** and **D** show the correlation for the switching costs, short and long CTI, respectively

By contrast, there was a significant association between the FPN-ROI homotopic connectivity and mixing costs with both short and long CTI (Fig. 7, Panels A, B), with the latter surviving multiple comparison correction (*r*_s_ = 0.493; *p* = 0.001). A negative trend was observed between FPN-homotopy and switching costs with long CTI, although not surviving correction for multiple comparisons (*r*_s_ = 0.364; *p* = 0.015; Fig. 7, Panel D). No significant association was found for FPN-homotopy and switching costs with short CTI (Fig. 7, Panel C). Overall, this analysis showed that the correlation between homotopy and cognitive measures was significant and survived correction for multiple comparisons in FPN regions, especially for mixing costs.

**Fig. 8:**
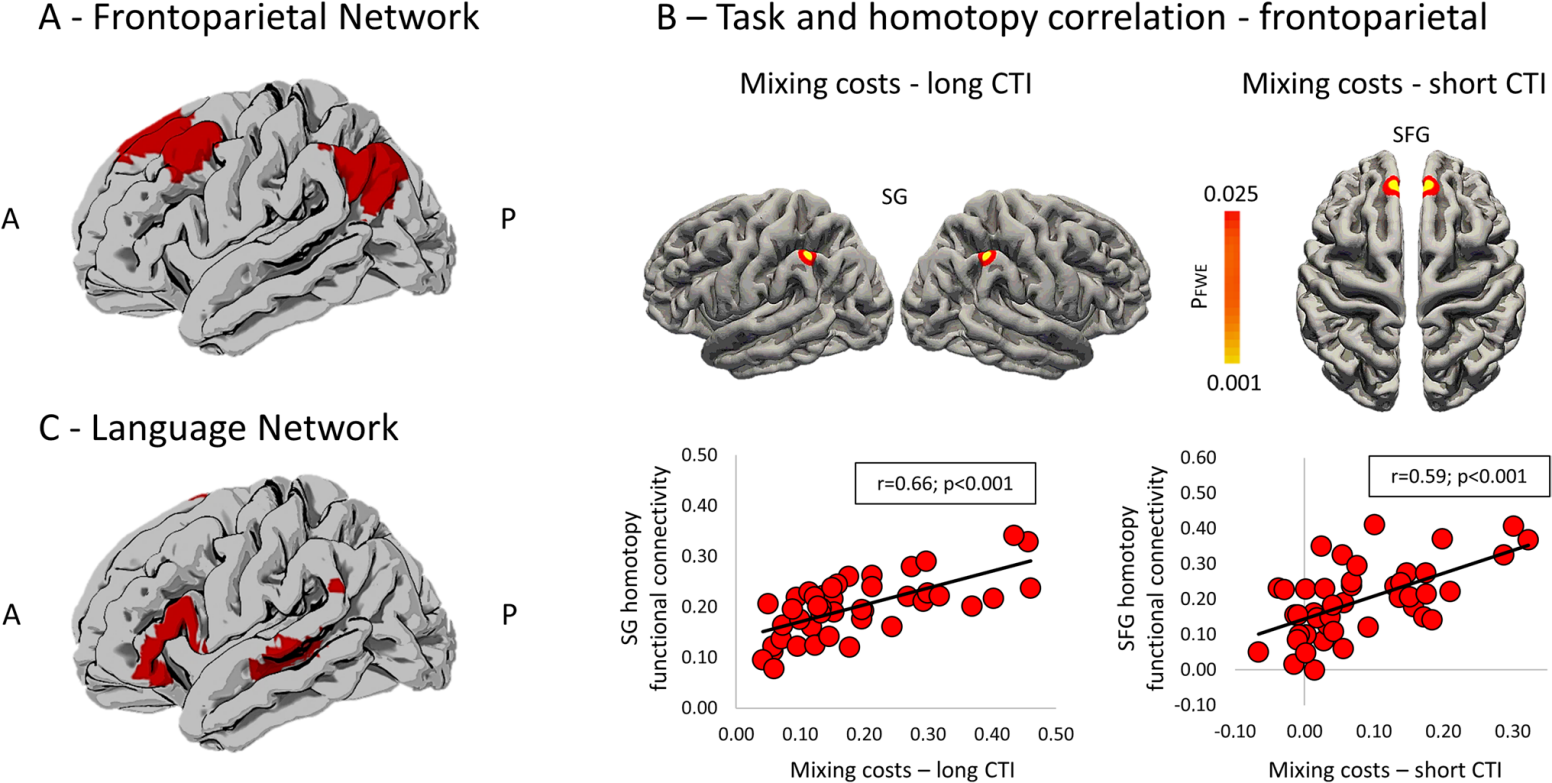
Fronto-parietal region-of-interest from group ICA registered to the asymmetrical MNI template and mapped to the fsaverage surface (**A**). Clusters showing significant positive association were reported for both mixing costs in the long CTI, mapping to the supramarginal gyrus (SG), and mixing costs in the short CTI, mapping to the superior frontal gyrus (SFG). Significant results are shown at *p* < 0.025 FWE-corrected (Panel **B**, top). Post hoc analysis between mixing costs and averaged homotopy within significant clusters from voxel-wise analysis are shown in Panel **B**, bottom. No significant association was reported between executive tasks and homotopy properties of the language network shown in Panel **C**. *A* anterior, *P* posterior

We further investigated the association between homotopy and FPN-ROI with a voxel-wise level analysis. Clusters showing a significant positive association between homotopy and mixing costs were reported for each CTI. Specifically, for the long CTI, mixing costs were positively related to homotopy in a FPN cluster mapping to the supramarginal gyrus, while for the short CTI, the mixing costs were linked with homotopy values of the superior frontal gyrus (Fig. 8; Table 2). The plots of the distribution of correlation values between behavioral performance and averaged homotopy of these clusters (assessed with Pearson’s correlation) could be appreciated in Fig. 8. In a *post-hoc* analysis, we investigated whether other frontal regions showed a significant correlation with the switching task performance. To this aim, the group FPN map from GIFT was thresholded at a threshold of *z* = 1, which allowed the inclusion of larger clusters mapping to the dorsolateral prefrontal cortex and in the temporal gyrus. We applied the same statistical model with a more lenient threshold (TFCE level *p* < 0.001, uncorrected). This analysis showed positive homotopy-behavior correlations in the same clusters reported in the main analysis (Supplementary Figure S2). Finally, homotopy functional connectivity within the language network (control analysis) did not show significant associations at the voxel-wise level with either mixing or switching costs, as expected.

**Table 2:** Coordinates showing a significant positive association between homotopic functional connectivity and behavioral measures of mixing costs for long and short Cue-to-Target-Interval (CTI)

Behavioral measure	Region	Peak voxel coordinates			Peak MNI coordinates			Brodmann area	Cluster size (#voxels)	FWE *p*
		*x*	*y*	*z*	*x*	*y*	*z*			
Mixing costs, Short CTI	Superior frontal gyrus	|30|	58	42	|4|	30	53	8	27	0.014
Mixing costs, Long CTI	Supramarginal gyrus	|14|	28	40	|46|	− 48	44	40	29	0.005

The location of significant clusters is referred to in terms of the coordinates of peak *p* value in the ICBM 2009a Nonlinear symmetric template voxel space (3 voxel size isotropic resolution; neurological orientation). Coordinates are reported also in MNI after registering the clusters in the 2 mm MNI template. Clusters are reported in the left hemisphere for simplicity but refer to homotopy measures computed between both hemispheres. *pFWE* *p* value after family-wise error correction

### Functional connectivity strength and cognitive performance

**Fig. 9:**
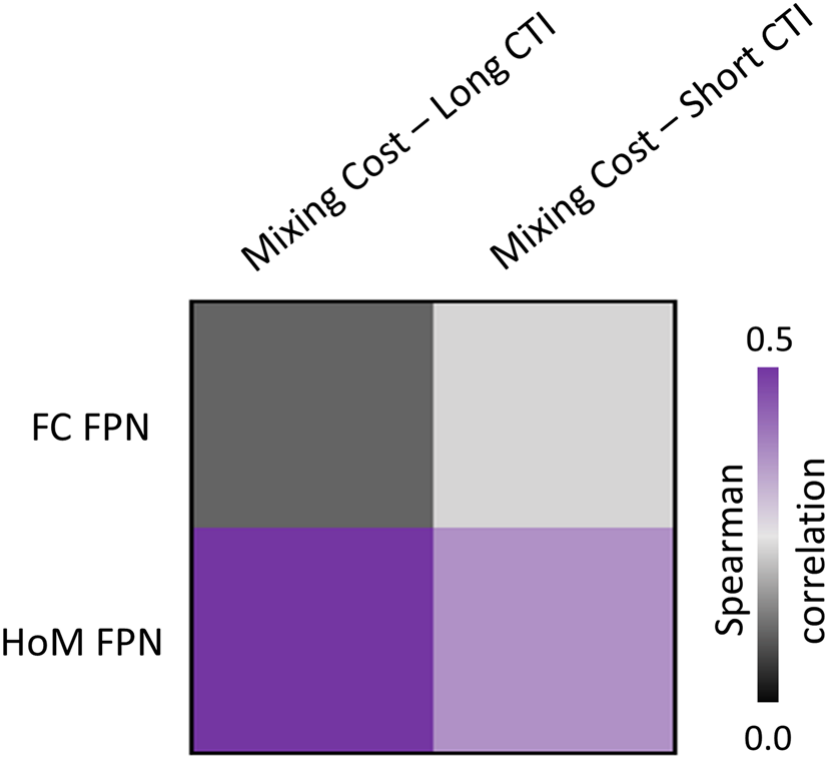
Frontoparietal network (FPN) properties correlated with the mixing cost performance, for both short and long Cue-to-Target Intervals (CTIs). The correlation between brain organization and cognitive tasks was numerically higher for the homotopy features for both CTIs. *FC *functional connectivity, *HoM *homotopy

In contrast to the homotopy results, we did not find a significant association between functional connectivity strength and task-switching performance. At the ROI level, the connectivity strength of the FPN was not linked with either mixing costs (CTI long: *r*_s_ = 0.117; *p* = 0.449; CTI short: *r*_s_ = 0.216; *p* = 0.158; see Fig. 9), or switching-costs (CTI long: *r*_s_ = − 0.146; *p* = 0.343; CTI short: *r*_s_ = − 0.292; *p* = 0.054). This result was in line with the voxel-wise analysis, where we did not observe voxels expressing a significant relationship between connectivity strength and task performance, for any of the contrasts investigated. To ensure that these results were not threshold-dependent, we performed this correlational analysis without applying a threshold to the FPN maps. The same null results were confirmed for both ROIs (mixing costs: CTI long: *r*_s_ = − 0.098; *p* = 0.526; CTI short: *r*_s_ = − 0.294; *p* = 0.053; switching costs: CTI long: *r*_s_ = 0.109; *p* = 0.483; CTI short: *r*_s_ = 0.232; *p* = 0.130) and voxel-wise approaches.

### Functional and behavioral reliability

**Fig. 10:**
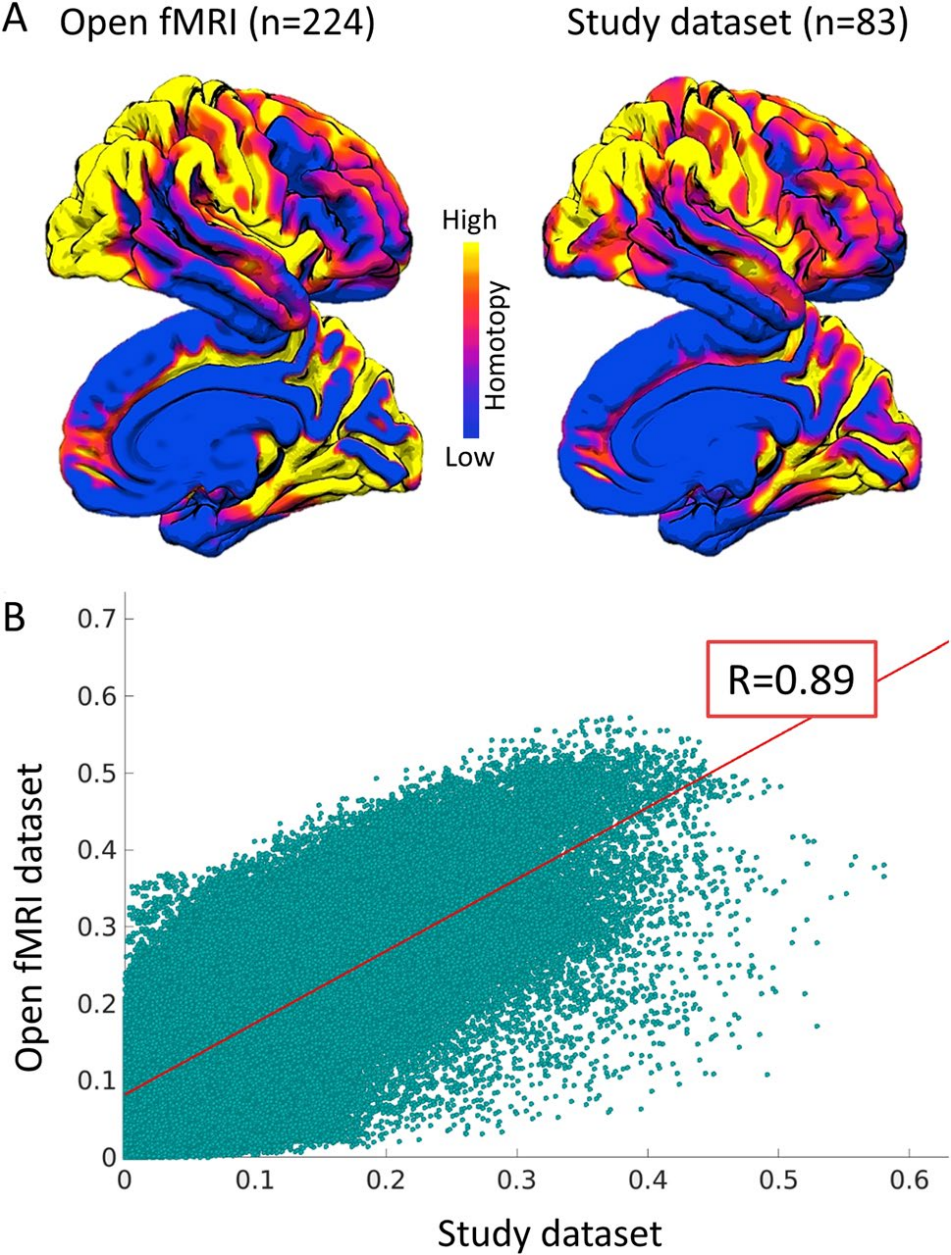
**A** Mean homotopy maps from the OpenfMRI dataset and the study dataset (scaled at different *Z* Fisher values). **B** Spatial voxel-wise correlation between the open fMRI dataset and dataset maps of the current study

As shown in Fig. 10 (panel A), the homotopy maps from the PIOP2 cohort and our study dataset were similar. The spatial cross-correlation between mean (*Z*-Fisher) maps was *r* = 0.89 (Fig. 10; panel B). A similar cross-correlation value was reported when comparing one-sample t-maps, with *r* = 0.92, suggesting that homotopy features are highly reproducible among independent datasets.

For the behavioral measures, reliability of mean RTs for the six trial type-by-CTI combinations was: 0.96 (range of Pearson’s correlation coefficients: 0.91–98) for pure trials at short CTI; 0.96 (range: 0.92–0.99) for pure trials at long CTI; 0.92 (range: 0.80–0.97) for repeat trials at short CTI; 0.93 (range: 0.79–0.98) for repeat trials at long CTI; 0.91 (range: 0.81–0.96) for switch trials at short CTI; 0.91 (range: 0.75–0.97) for switch trials at long CTI. Median correlations for switching costs were 0.89 (range: 0.71–0.96) at short CTI and 0.88 (range: 0.63-0.97) at long CTI. Median correlations for mixing costs were 0.81 (range: 0.59–0.94) at short CTI and 0.87 (range: 0.61–0.96) at long CTI.

## Discussion

This study was focused on understanding the relationship between task-switching performance and functional FPN properties. Overall, the present results show that FPN homotopy is associated with cognitive performance in a task-switching paradigm. Specifically, higher homotopy connectivity was linked with worse cognitive outcomes. This result echoes previous studies suggesting that higher homotopy might represent a proxy of cognitive impairment, as observed in several neurological conditions (Guo et al. 2013; Zhang et al. 2015). As expected, the association was significant for the FPN, whereas the relationship between whole-brain homotopy organization and task-switching performance did not survive multiple comparison correction. These findings suggested that homotopy connectivity of brain regions belonging to the FPN are linked with executive processes underlying performance on this complex task, in line with the assumption that this network represents a critical hub for cognitive control in a goal-driven manner (Marek and Dosenbach 2018). Notably, the relationship between FPN functional connectivity (ICA map) and cognitive performance was not significant, suggesting that FPN hemispheric specialization might be a more sensitive proxy of higher cognitive abilities compared to measures of within-network connectivity strength.

Interestingly, our results showed a differential association of homotopy in the superior frontal gyrus and the supramarginal gyrus with mixing costs for the short and long CTIs, respectively. Notably, these relationships seem specific to FPN areas, as no association at the voxel-wise level was present between behavior and homotopy in hubs of the control language network. These findings might suggest that different sub-processes are hosted within the FPN homotopy gradient during task-switching, as already shown elsewhere (e.g., Muhle-Karbe et al. 2014). In particular, short CTIs make task-switching-related processes more demanding, as reported in previous literature for switching costs (Koch 2001; Monsell 2003; Arrington and Logan 2004; Petruo and Beste 2021), and demonstrated by our behavioral data analysis also for mixing costs (cf., Manzi et al. 2011). Mixing costs for the short CTI were indeed more than twice as big as those for the long one (186 vs. 81 ms, see Table 1). While variable foreperiod effects (e.g., Niemi and Näätänen 1981; Vallesi et al. 2014) could have surely contributed to this performance difference between the two CTIs, the short CTI condition conceivably entails more uncertainty and higher demands on goal maintenance processes reflected by mixing costs (Cooper et al. 2015). Control of higher-level goal representations is hosted in rostral medial premotor/prefrontal regions (e.g., Taren et al. 2011; Korb et al. 2017; Badre and Nee 2018), which is consistent with the locus of the homotopy correlation effect with short CTI mixing costs.

Mixing costs with long CTI (1200 ms) instead positively correlated with homotopy in the supramarginal gyrus. Task-switching performance with long CTI probably relies less on task-goal maintenance and more on transforming well-prepared abstract task-sets to specific sensori-motor actions during task implementation, which is a function attributed to superior parietal regions (Bunge et al. 2002; Brass et al. 2005). These results are consistent with those of a recent TMS study (Muhle-Karbe et al. 2014). In that study, it was found that inhibitory TMS over the intra-parietal sulcus (MNI: − 34, − 56, 43), close to the peak found here in the supramarginal gyrus (|46| − 48 44), disrupted the ability to update response-specific sets, but not general task goals, that were perturbed only when the pulse was delivered on this parietal region closer in time to the response execution. Furthermore, consistent with our results, parietal regions (Brodmann area 40) have been proposed to be involved in the preparation of possible switches of response sets during the foreperiod (Wolff et al. 2018).

A parietal contribution to motor processes during task-switching performance has been also reported in a recent electroencephalographic (EEG) study that, by applying signal decomposition and source reconstruction of EEG data, showed response-related parietal modulations for switching trials (Petruo and Beste 2021). Although a direct comparison between this previous study and ours is hampered by the fact that our results concerned the mixing costs, whereas their focus was on the switching cost, it is interesting to note that both studies point toward a role of motor remapping processes during task switching performance. Of note, it should also be considered that response processes related to parietal activations have been shown to be modulated by age which, as introduced earlier, is critical in shaping homotopic connectivity (Dilcher et al. 2021). Indeed, intrinsic connectivity undergoes more maturational changes over the lifespan in multimodal associative parietal areas, such as the precuneus, than in other brain areas, such as in the default mode network (Yang et al. 2014; Gilmore et al. 2015).

Based on previous literature (Kim et al. 2012; Muhle-Karbe et al. 2014; Vallesi et al. 2015; Ambrosini and Vallesi 2016), one could also expect that more lateral prefrontal regions would be important for mixing costs and, thus, show homotopy-related correlations with switching and mixing costs. However, in a *post-hoc* analysis including more lateral prefrontal regions in the definition of the FPN, we did not find additional clusters showing homotopy-behavior correlation (see Supplementary Figure S2).

It is worth noting that no relationship between homotopy and switch costs emerged. At first glance, this null finding could be surprising given that previous studies reported a role for functional hemispheric asymmetries in predicting switching costs (Ambrosini and Vallesi 2016). However, further work investigating brain-behavior correlations also failed to observe correlations for switching, but not for mixing, costs (e.g., Treit et al. 2014; Vallesi et al. 2016). Although non-significant effects are always difficult to interpret, this might be due to the fact that at least partially similar neural and cognitive mechanisms are implicated in switch and repeat trials, even if at a different extent, especially when the two trial types occur equiprobably as in the present study (Braver et al. 2003; Crone 2006; Ruge et al. 2013; Vallesi et al. 2015). Further studies manipulating the proportion of switch and repeat trials are needed to sort this issue out.

Finally, the reported general homotopy pattern is consistent with previous literature showing higher values of homotopic connectivity in sensory-motor regions and lower values in associative cortices, such as the prefrontal cortex (Garcia-Tabuenca et al. 2018; Mancuso et al. 2019; Jin et al. 2020; Zuo et al. 2010). This pattern shows several commonalities with the principal functional gradient recently suggested by Marguiles and colleagues (2016). Brain regions showing similar connectivity patterns are grouped together along this functional axis. More specifically, the gradient reflects the main axis of connectivity variance spanning from unimodal (sensory) to transmodal (associative) brain areas (Margulies et al. 2016). It has been suggested that the principal functional gradient might underlie differences in the underlying cortical organization, represented by a bottom-up organization of unimodal regions and a denser top-down interconnectivity of transmodal regions, enabling a more flexible and integrated response to a different type of stimuli (Mesulam 2012; Vázquez-Rodríguez et al. 2019). The functional homotopy pattern might follow this hierarchical cortical organization. This assumption might be supported by the brain-behavioral results. Clusters showing a significant relationship with task performance were positioned at the two ends of the topographical rostral-caudal gradient of the transmodal integrative hubs reported by Marguiles and colleagues (2016), involving the superior frontal gyrus and angular/supramarginal gyrus. These regions are both distant from unimodal systems and have been suggested to act as hubs of integration across multiple sensory modalities (Margulies et al. 2016). Further studies are necessary to investigate this assumption and the underlying relationship between homotopy and functional gradients.

### Strengths and limitations

This study has both strengths and limitations. The main merit is that the still poorly understood relationship between executive functions and brain homotopy was investigated using state-of-the-art tools for assessing both cognitive functions and brain connectivity. Second, a data-driven approach (ICA) was implemented to determine FPN hubs, in which the relationship between functional homotopy and task-switching performance was assessed. Data-driven connectivity-based parcellation might increase functional connectivity representation compared to existing atlases (Ren et al. 2019).

Among the limitations, we acknowledge that, since our focus was on young adults with a homogeneous age range, our results cannot be generalized to the whole lifespan. In this regard, our work could be considered as an ideal starting point to further investigate whether the relationship between task-switching performance and functional homotopy is also evident in other age groups.

## Conclusions

In conclusion, the present findings extend previous literature on the relationship between brain functional homotopy and cognitive efficiency to task-switching performance, and show a dissociable role of homotopy in different fronto-parietal areas depending on task demands. Thus, brain homotopic connectivity does not just appear to be a mere epiphenomenon emerging from connectivity computations, but it is shown to underlie functional complexity meaningfully linked with higher cognitive functions.

## Supplementary Information

Below is the link to the electronic supplementary material.

Supplementary file1 (DOCX 657 KB)

## Data Availability

The datasets used for the current study are available from the authors upon reasonable request.

## Code availability

The codes used for all the analyses are available upon reasonable request from any qualified investigator.
