# Supplementary material for: Fronto-parietal homotopy in resting-state functional connectivity predicts task-switching performance
Source: Brain Struct Funct. 2021 Jun 9;227(2):655–72. doi: 10.1007/s00429-021-02312-w (PMC8843912; doi:10.1007/s00429-021-02312-w)

**Supplementary Material**

**Fronto-parietal homotopy in resting-state functional connectivity predicts task-switching performance**

Antonino Vallesi, Antonino Visalli, Zeus Gracia-Tabuenca, Vincenza Tarantino, Mariagrazia Capizzi, Sarael Alcauter, Dante Mantini, Lorenzo Pini

**Supplementary Methods**

*MRI data acquisition*

Resting-state-fMRI, and structural MRI data for an additional independent sub-sample of 21 young participants (without task-switching data) were acquired on a 3T Ingenia Philips whole body scanner (Philips Medical System, Best, The Netherlands) equipped with 32-channel head-coil (San Camillo IRCCS Hospital, Venice, Italy). The following sequences and parameters were used: i) 200 T2*-weighted echo-planar image (EPI) volumes for rsfMRI data analysis (TR/TE=2000/30ms; 39 axial-slices with ascending acquisition; voxel size: 3x3x3 mm; flip angle=76°; acquisition matrix: 84x84); ii) structural 3D T1-weighted image (TR/TE=8.2/3.8 ms; voxel size: 1x1x1 mm; FA: 8; acquisition matrix: 256x256). This additional dataset was approved by the Comitato Etico per la Sperimentazione Clinica di Venezia e IRCCS San Camillo (Prot # 2017.07).

**Supplementary Table**

*Supplementary Table S1*. Codes of the 224 subjects retrieved from the Population Imaging of Psychology dataset (PIOP2) released within the Amsterdam Open MRI Collection, used for the reliability analysis. Only subjects with both structural MRI and resting-state fMRI data available were selected (see main text for details).


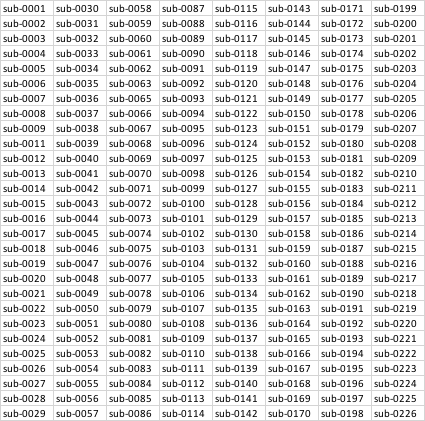


**Supplementary Figures**

*Supplementary Figure S1*. Homotopy maps in the sample of participants who performed both functional MRI and the task-switching tests (n = 44). Mean Z-fisher transformed maps are displayed over the left fsaverage surface. Red colors: higher homotopy functional connectivity.


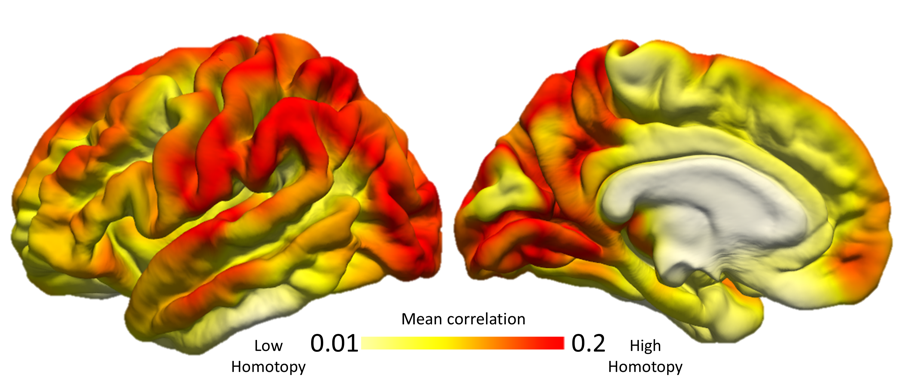


*Supplementary Figure S2*. Significant voxel-wise positive association between behavioral measures and homotopy within fronto-parietal network region-of-interest (red clusters; panel A) obtained with a more lenient threshold z=1. Inclusion of a higher portion of FPN showed the same pattern of relationships between task and homotopy in the prefrontal (mixing costs-short CTI) and parietal (mixing costs-long CTI) nodes (Panel B) as the main analysis. Results are reported at *p <* 0.0005, uncorrected.


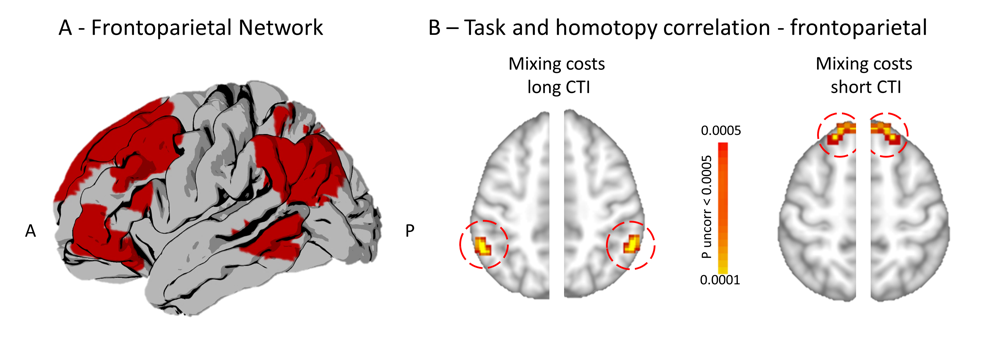

Supplement: Supplementary file 1 — Supplementary file1 (DOCX 657 KB) [file 429_2021_2312_MOESM1_ESM.docx]
